# Electrospun Naringin-Loaded Fibers for Preventing Scar Formation during Wound Healing

**DOI:** 10.3390/pharmaceutics15030747

**Published:** 2023-02-23

**Authors:** Erika M. Tottoli, Laura Benedetti, Enrica Chiesa, Silvia Pisani, Giovanna Bruni, Ida Genta, Bice Conti, Gabriele Ceccarelli, Rossella Dorati

**Affiliations:** 1 Department of Drug Sciences, University of Pavia, 27100 Pavia, Italy; 2 Department of Public Health, Experimental Medicine and Forensic, University of Pavia, 27100 Pavia, Italy; 3 CHT Center for Health Technologies, University of Pavia, 27100 Pavia, Italy; 4 Department of Otolaryngology, IRCCS Policlinico S. Matteo, 27100 Pavia, Italy; 5 Department of Chemistry, Physical-Chemistry Section, University of Pavia, Via Taramelli 16, 27100 Pavia, Italy

**Keywords:** hypertrophic scar, electrospinning, wound healing, complex wounds

## Abstract

Hypertrophic scars (HTSs) are aberrant structures that develop where skin is injured complexly and represent the result of a chronic inflammation as a healing response. To date, there is no satisfactory prevention option for HTSs, which is due to the complexity of multiple mechanisms behind the formation of these structures. The present work aimed to propose Biofiber (Biodegradable fiber), an advanced textured electrospun dressing, as a suitable solution for HTS formation in complex wounds. Biofiber has been designed as a 3-day long-term treatment to protect the healing environment and enhance wound care practices. Its textured matrix consists of homogeneous and well-interconnected Poly-L-lactide-co-poly-ε-caprolactone (PLA-PCL) electrospun fibers (size 3.825 ± 1.12 µm) loaded with Naringin (NG, 2.0% *w*/*w*), a natural antifibrotic agent. The structural units contribute to achieve an optimal fluid handling capacity demonstrated through a moderate hydrophobic wettability behavior (109.3 ± 2.3°), and a suitable balance between absorbency (389.8 ± 58.16%) and moisture vapor transmission rate (MVTR, 2645 ± 60.43 g/m^2^ day). The flexibility and conformability of Biofiber to the body surfaces is due to its innovative circular texture, that also allow it to obtain finer mechanical properties after 72 h in contact with Simulated Wound Fluid (SWF), with an elongation of 352.6 ± 36.10%, and a great tenacity (0.25 ± 0.03 Mpa). The ancillary action of NG results in a prolonged anti-fibrotic effect on Normal Human Dermal Fibroblasts (NHDF), through the controlled release of NG for 3 days. The prophylactic action was highlighted at day 3 with the down regulation of the major factors involved in the fibrotic process: Transforming Growth Factor β1 (TGF-β1), Collagen Type 1 alpha 1 chain (COL1A1), and α-smooth muscle actin (α-SMA). No significant anti-fibrotic effect has been demonstrated on Hypertrophic Human Fibroblasts derived from scars (HSF), proving the potential of Biofiber to minimize HTSs in the process of early wound healing as a prophylactic therapy.

## 1. Introduction

Complex wounds are defined as wounds that fail to proceed through the normal phases of wound healing in an orderly and timely manner [1,2,3,4]. Pathomechanisms which lead to the formation of hypertrophic scars (HTSs) are not yet completely understood, and this has led to various therapeutic approaches aimed at constraining HTSs; however, there is no standard and specific evidence-based treatment protocol [5]. According to their unpredictability of onset, the HTS etiology is just based on case studies and the literature; this pathological form of scarring occurs in complex wounds with an incidence of 35% after surgery and up to 80% following burn injuries, and depends on age, genetics, and ethnicity of the patient [6]. 

Various conventional dressing materials are used for treating complex wounds, the most common being a combination of paraBin-impregnated gauze and an absorbent cotton wool layer [7]. However, these conventional dressings are not able to constrain HTS formations, tend to adhere to the wound bed, and need frequent change procedures that alter the epithelialized surfaces and delay healing [8]. An advanced dressing must promote the fluid absorption, while maintaining a correct humidity gradient in the wound site, to encourage granulation and assist epithelialization. Moreover, an advanced dressing should provide the modulation of the inflammation process that causes a delay in the healing process, and lead in this way to the formation of HTSs and other pathological structures. To promote a rapid and physiological healing, an advanced medicated dressing should be a consistent bacterial barrier to prevent infection entering the wound or being transmitted from the wound [8]. 

In this context, nano-fiber dressings have great potential for providing most of the ideal dressing features [9]. In addition, the electrospun dressing can imitate the extracellular matrix; regulate the cellular responses of skin, including proliferation, migration, and differentiation, drastically reducing the healing time of injuries and facilitating the healing of complex injuries. Polymeric electrospun wound dressings have been extensively researched, and the findings have been discussed in various review publications [10,11,12]. Electrospun wound dressings have been extensively investigated as drug delivery systems containing several antibacterial agents (formyl phenylboronic acid, zinc oxide and silver nanoparticles) intended to reduce the risk of infections in chronic wounds [12,13,14,15]; to the best of our knowledge, no papers in the literature considered electrospun dressing as preventive therapeutic treatment of HTSs by delivering antifibrotic agent.

The main goal of this work was to improve effectiveness of Biofiber with the feature of preventing the appearance of scars after injuries and offer a suitable solution to fill the lack of treatments in this field. Biofiber has been conceived as a biodegradable electrospun wound dressing to prevent scarring in deep partial thickness burns [16]. It provides a valuable platform for managing exudate by creating suitable conditions for physiological healing [17]. In this research paper, an ancillary medicinal substance (NG) was incorporated into fibers to prevent scarring during the burn healing process. Much research has demonstrated the biological effects of NG as an antioxidant, anti-inflammatory, and anti-apopsis; additionally, evidence has been shown that NG may attenuate hepatic fibrosis in rats suppressing TGF-β1 [18,19].

## 2. Materials and Methods

### 2.1. Materials

PLA-PCL 70:30, (Resomer LC 703 S, Mw 160 kDa, Tg 37 °C) was purchased from Evonik Nutrition and Care (Darmstadt, Germany) and dissolved in 20% (*w*/*v*) of dichloromethane (DCM, CH_2_C_l2_), analytical grade 99.9%, Mw 84.93 Da (Carlo Erba SpA, Milan, Italy). To allow the correct solubilization of polymer, the system was maintained under magnetic stirring at 100 rpm in an ice bath. Naringin (NG) C_27_H_32_O_14_, high chemical grade 95% (Sigma Aldrich, Milan, Italy) was solubilized in 8% *v*/*v* of N,N-dimethylformamide (DMF, C_3_H_7_NO) analytical grade 99.8%, Mw 73.09 Da at (Carlo Erba Spa, Milan, Italy). NG solubilization was carried out by magnetic stirring in an ice bath to prevent solvent evaporation. The NG suspension was added, drop by drop, to the polymeric solution, and it was maintained under magnetic stirring for 30 min in an ice bath.

### 2.2. Polymer Solution Preparation and Characterization

The rheological properties of the polymer solutions were performed using the Rotational Rheometer Malvern Kinexus Pro+ equipped with a CP4/40 flat cone geometry (40 mm diameter, 1° cone angle).

The amplitude sweep tests were performed at 32 °C, at a constant frequency of 1 Hz and shear stress (σ) ranging from 10^−2^ to 10^5^ Pa to determine the linear viscoelastic region (LVER). Shear rate ramp analyses were performed in the LVER range at 32 °C. For the viscosity concentration assessment, the rheological behavior was investigated on a wide range of PLA-PCL concentration ranging from 0.1 to 20.0% *w*/*v*, the polymer solution specific viscosity (*η_sp_*), which considers the complex solvent system contribution to the overall viscosity, was calculated using following Equation (1).

(1) $$\eta_{sp}=\left(\eta_{in}-\eta_{s\;}\right)/\eta_{s}$$

where *η_in_* is the intrinsic viscosity of the polymer formulation, while *η_s_* is the viscosity of the complex solvent system corresponding to 0.536 mPa.

### 2.3. Preparation of Textured Dressing

Texturized fiber dressings were prepared using electrospinning Nanon-01A (MEEC Instruments, ltd., Ogorishi, Fukuoka, Japan) according to patent WO2021064673. The process was carried out at 30 ± 2 °C, RH 25 ± 5%. The electrospinning parameters were set up as specified below: spinneret speed and width (100 mm/s and 80 mm), cleaning frequency (30 s), voltage 20 kV, flow rate 0.6 mL/h), nozzle diameter (18G), and electrospinning time 16 min. 

### 2.4. Advanced Dressing Characterization

The advanced dressing characterization analysis was carried out comparing Biofiber and placebo (PL) prototypes to a non-textured electrospun internal control (1P) and two commercially available advanced medicated dressings namely Mepilex Lite^®^ (Mölnlycke Health Care AB, Goteborg, Sweden) and Biatain^®^ Alginate (Coloplast S.p.A, Bologna, Italy). Mepilex Lite^®^ is a polyurethane foam-based advanced dressing designed for the treatment of acute and chronic wounds with medium exudation. Biatain^®^ Alginate is a highly absorbent alginate dressing for highly exuding wounds; it has a higher absorption than other alginate and hydrofiber dressings [16].

#### 2.4.1. Morphology Characterization by Scanning Electron Microscopy (SEM)

Morphological characterization was carried out on placebo (PL) and Biofiber electrospun matrices by scanning electron microscopy (SEM). Zeiss EVO MA10 apparatus (Carl Zeiss, Oberkochen, Germany) was used to characterize the matrix morphology in terms of size, shape, and orientation of fibers. Dressing prototypes were cut appropriately into squares of 0.3 × 0.3 cm; each sample was fixed on carbon supports and it was covered with a gold layer. All samples were observed at different magnifications (50×, 500×, 1.0K× and 5.0K×) and accelerated voltages (20 kV) in high vacuum at room temperature. An analysis was performed to determine the matrix thickness and relative differences between its no-textured and round textured areas (700×). All SEM images were analyzed by ImageJ software, *n* = 50.

#### 2.4.2. Wettability Evaluation

The wetting behavior of each prototype was measured on electrospun circular samples (2 cm diameter) at room temperature (22 ± 3 °C) and relative humidity of 36%. Measurements were conducted using the contact angle meter (Kyowa Interface Science, made in Japan, model: DMe-211Plus) with FAMAS software for data processing.

Simulated Wound Fluid medium (SWF) was selected as the hydrophilic solution to test the dressing wettability, the contact time between the drop and the sample was set at 9 s and the SWF falling volume was 1 µL.

To formulate SWF solution (100 mL), 50 mL of Bovine Foetal Serum mycoplasma and virus secerned (FBS 50:50 *v*/*v*; Immunological Science, Rome, Italy) was added to 50 mL of Maximum Recovery Diluent (MRD 9.5 g/L, Sigma Aldrich, Milan, Italy). The results are expressed as average ± standard deviation (*n* = 3).

#### 2.4.3. Fluid Handling Capacity

The fluid handling capacity (FHC), expressed as the sum of dressing absorbency and moisture vapor transmission rate (MVTR) indicates the bandage’s ability to control exudate [20]. These parameters were evaluated following the European standard BS EN 13726–1: Test Methods for Primary Wound Dressings. The data are expressed as mean ± standard deviation (*n* = 3).

##### Absorbency of Dressing

The dressing absorbency was measured through a gravimetrical analysis using an analytical balance. The electrospun circular samples (2 cm diameter) were soaked in SWF and incubated at 34 ± 2 °C, 30% RH for 24 h. At scheduled experimental times, samples were removed from the medium and fluid exceedance was drained for 60 s. Dressing absorbency was calculated following Equation (2). The results are expressed as average ± standard deviation, *n* = 3.

(2) $$Absorbency\;\%=\left(W_{wet}-W_{dry}\right)/W_{dry}$$

##### Moisture Vapor Transmission Rate (MVTR)

To determine the moisture permeability of Biofiber prototypes, the MVTR was measured according to the ASTM E 95-96 (1995) guideline: American Standard Test Methods for Water Vapor Transmission of Materials [21,22]. Briefly, samples were cut into a disc (2 cm of diameter), weighed, and conditioned for 24 h into constant climate chamber HPP (Memmert GmbH + Co. KG, D-91186 Büchenbach, Germany) at 34 °C and 11% RH to achieve moisture content equilibrium. Subsequently conditioned samples were weighed and mounted on the mouth of a cylindrical cup (1 cm^2^ opening area) containing distilled water (40 mL). After weighing the whole system, samples were placed into a climate chamber (34 °C and 11% RH). Later 24 h, all samples were weighed to calculate the water mass loss. Results were calculated with Equation (3), and expressed as average ± standard deviation, *n* = 3.

(3) $$MVTR\;\left(g\;cm^{3}\;day^{-1}\right)=\Delta m/\left(A*t\right)$$

where *m* is the water mass loss (mg), *A* is the area of sample (cm^3^) and *t* refers to the test period expressed in day, *n* = 3.

##### Vertical Wicking

To perform the vertical wicking analysis, dried sample strips (5.0 × 40 mm) were obtained using a blade and gently placed vertically in SWF up to 10 mm length. The testing time was set up at 60 s, at the end of which the vertical wicking was determined in mm with a ruler.

Vertical wicking was performed in triplicate and results are expressed as average ± standard deviation (*n* = 3).

#### 2.4.4. Mechanical Properties

The tensile mechanical properties of dressing prototypes were examined by a mechanical tester machine for monoaxial tensile tests (Mark-10 ESM303, Force Gauge Model MI5-5, G1013; Copiague, NY 11726, USA), software MESUR gauge Plus (Copiague, NY 11726, USA). The assessments were conducted following ASTM D882 (2002) guideline: American Society Standard Test Method for Tensile Properties of Thin Plastic Sheeting (<1 mm). Dog bone-shape prototypes 80 × 10 × 4 mm were obtained through a calibrate die-cutting machine; measures were made at a constant tensile deformation rate of 15.1 mm min^−1^. The analysis was useful to determine the elongation-at-break, ultimate tensile stress (UTS), breaking point, yield strength and Young’s modulus.

The tensile test was carried out on Biofiber prototypes, Mepilex lite^®^ and Biatain^®^ Alginate samples in dry conditions and after incubation at 34 °C in SWF at schedule experimental times (24–72 h), to simulate the mechanical behavior of membranes in contact with the wound exudate. The results are expressed as the differences of the average between dry and wet condition ± standard deviation, *n* = 10.

#### 2.4.5. Dressing Integrity

The Biofiber advanced electrospun dressing was designed to perform a sustained activity over three days on the wound bed. To achieve this purpose, it is necessary that the dressing maintains its structure during the treatment time. The integrity of the fibrous bandages is crucial to avoid both contamination of the wound bed by bandage debris and loss of their absorption function [23].

##### Mass Loss

The in vitro mass loss evaluation was carried out to assess the electrospun prototype degradation when involved in an experimental protocol that mimics the wound environment. Similar to dressing absorbency, samples (2 cm diameter, 10.7 ± 3.5 mg) were incubated for 5 days in SWF, and at scheduled times, were recovered and freeze-dried (Lio-5P, Cinquepascal, Italy) at −48 °C and 0.4 mbar. The mass loss was determined by gravimetric analysis through Equation (4).

(4) $$ML\;\left(\%\right)=\left[\left(w_{t}-w_{0}\right)/w_{0}\right]*100$$

*w_t_* is the weight of sample after freeze-drying, *w*_0_ is the initial weight of sample. The results are expressed as average ± standard deviation, *n* = 3.

##### GPC Analysis

Gel Permeation Chromatography (GPC) made up of an injector, three columns (Plgel 5 µm 500 Å 300 × 7.5 mm, PL Aquagel-OH MIXRD-H 8 µm, 1 × 10^3^ Å, and Phenogel 5 µm 1 × 10^4^ Å both 300 × 7.5 mm, a pre-column (Plgel 5 µm 50 × 7.8 mm), and a refractive index detector, was selected for evaluated dressing stability after 5 days of incubation in SWF at 34 °C (simulated conditions), *n* = 3.

The degradation entity was analyzed as a variation of weight average molecular weight (*Mw*) over time. The calibration curve was obtained through several polystyrene powder standards (4490, 8450, 19,760, 38,100, 70,950, 143,400, 316,500 Da). The data are expressed as mean ± standard deviation (*n* = 3).

### 2.5. Drug Content and Encapsulation Efficiency Determination

The NG drug content (DC) and encapsulation efficiency (EE%) were determined cutting the dressing sample in three distinctive square parts (1 × 1 cm); each sample was weighted, put in a glass vial, and dissolved in DCM (1 mL).

**Figure 1:**
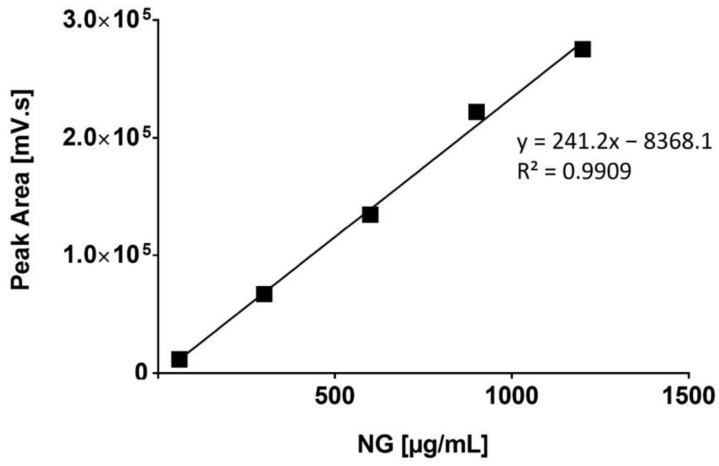
Calibration curve of NG solutions of concentration range from 60 µg/mL to 1200 µg/mL. Standard deviations are not noticeable as <1.

The quantification protocol of NG was set up and validate by chromatographic analysis using a RI detector. An isocratic grade tetrahydrofuran (THF; Carlo Erba SpA, Milan, Italy) as mobile phase, with a flow rate set at 0.8 mL/min. The calibration curve was constructed using several NG powder standards ranging from 60 µg/mL to 1200 µg/mL (Figure 1). 

The chromatographic analysis was conducted in triplicate for each sample, and *DC* and *EE%* values were expressed as average ± standard deviation, *n* = 3. Drug content is expressed in µg/mg and was calculated by Equation (5), [24]:

(5) DC=NG actually in the sample (µg)/sample weight

The *EE%* was determined using Equation (6), wherein the theoretical mass of *NG* was determined by Equation (7), starting from the knowledge that the drug loaded in the polymeric solution is the 2% *w*/*w* of the polymer mass, expressed by sample mass. The data are expressed as mean ± standard deviation (*n* = 3), [24].

(6) EE %=[NG Actual mass/NG Theoretical mass]∗100

(7) Theoretical mass of NG (µg)=(2∗mass of the Sample)/100

### 2.6. In Vitro Release Study

An NG in vitro cumulative release test was performed in a time lapse of 72 h on Biofiber prototypes; this study was conducted to assess the ability of this electrospun dressing to promote a sustained and modulate release for three days, reducing the number of dressing removal practices and improve patient’s compliance. 

The samples were prepared by cutting each dressing in circular sample with 2 cm diameter; all samples were weighted (8.15 ± 0.54 mg) and then fixed into CellCrown™ (Sigma -Aldrich, Milan, Italy) inserts for a 12 multi-well (Sigma Aldrich, Milan, Italy). Following, they were dipped in 3 mL of Phosphate Buffered Saline 1X pH 7.4 (PBS 1X; Sigma Aldrich, Milan, Italy) and incubated in static conditions at 34 °C to simulate skin temperature. NG raw material (150 µg) was used to compare Biofiber with a no-controlled release system.

At scheduled times (2, 4, 6, 24, 48, and 72 h), 700 µL of PBS was withdrawn from each well and analyzed with the spectrophotometric 4 nm SBW spectrophotometer fitted with single 10 × 10 mm cuvette holder (Jenway model 6705 scanning UV–visible spectrophotometer) at 282 nm. 

**Figure 2:**
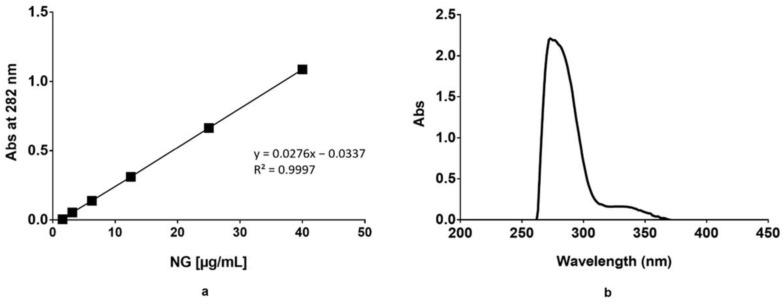
Calibration curve of Naringin solutions at different concentrations (3.125–40 µg/mL) measured at 282 nm (**a**), UV–Vis spectrum of NG standard solution in PBS 1X pH 7.4 (12.5 µg/mL) (**b**). Standard deviations are not noticeable as <0.01, plot a.

NG concentration was determined from a standard calibration curve prepared starting from a stock solution containing 1 mg/mL NG in PBS (Figure 2). The stock solution was diluted in a volumetric flask with PBS to obtain solutions of 3.125, 6.25, 12.5, 25, and 40 μg/mL of NG. Each standard solution was analyzed in triplicate, and each point of the calibration curve is the average of the three analyses. Standard deviations are not noticeable as <0.01.

### 2.7. Cell Culture

Adult normal human dermal fibroblast cells (NHDF, #LOCC2511) were bought from Euroclone S.p.A (Pero, Italy); Hypertrophic Scar-derived Fibroblasts Human (HSF; #HSF110 Lt Cheek) were isolated from the cheek skin of a 42-year-old male by CellResearch Corporation (Singapore). NHDF and HSF were cultured in Dulbecco’s Modified Eagle Medium 1% glutamine and 2% sodium pyruvate (DMEM; Sigma Aldrich, Milan, Italy) supplemented with 10% *v*/*v* FBS, 100 µg mL^−1^ penicillin, 100 µg mL^−1^ streptomycin (Immunological Science, Rome, Italy), and maintained at 37 °C with 5% CO_2_. All experiments were performed using cells cultured within six to seven passages.

### 2.8. Cytotoxicity Assay

NHDF and HSF cells were seeded at a density of 10^4^ cells/well in Cellstar^®^ 96-well cells culture plates (Avantor VWR, Milan, Italy) to establish Biofiber biocompatibility. Biofiber electrospun matrix was tested for its cytotoxicity [25,26]; untreated cells were used as the positive control (CTR+) and cells treated with the phenol solution (Phenol Liquified 85% Re; Carlo Erba, Milan, Italy) were used as a negative cell viability control (CTR−). 

Extracts of the electrospun dressings were prepared by incubating Biofiber round samples at different dimensions (0.25, 1, 2.25 cm^2^) in DMEM (2 mL) for 24–72 h at 37 °C with 5% CO_2_. All extracts were checked for their pH using 827 pH lab pH meter (Methron ion analysis Varese, Milan, Italy). pH values ranged between 7.4 and 7.5, indicating there was no massive release of PLA-PCL soluble degradation. 

NHDF and HSF were then incubated for 24 h with the respective extract, and the viability was evaluated following the ISO 10993-12 guideline: International Standardization Organization Biological Evaluation of Medical Devices [27]. After the incubation Thiazolyl Blue Tetrazolium Bromide assay (MTT, approx. 98% TLC, Sigma Aldrich, Milan, Italy) was performed. The MTT solution (0.5 mg/mL, Sigma-Aldrich, Milan, Italy) was added to cells for 3 h. Absorbance was measured at 570 nm with a microplate reader (HiPo MPP-96, OD plate (SIA Biosan, Riga, Latvia) with 690 nm as reference filter. 

The optical density value is directly proportional to the number of viable cells in the culture medium; to value the viability, Equation (8) was used:

(8) Viability %=(Abs Sample/Abs Control)∗100

The data are expressed as mean ± standard deviation (*n* = 9).

### 2.9. Live/Dead Staining

The morphological state of cells and their viability was evaluated by the Invitrogen LIVE/DEAD^®^ staining (Thermo Fisher Scientific, Milan, Italy). 

NHDF and HSF were seeded at a density of 5 × 10^4^ cells in Falcon 35 mm cell culture dishes (avantor VWR, Milan, Italy) and grown for 24 h in 2 mL DMEM for 24 h to allow cell stabilization after seeding; 500 μL of solution (1.5 mL of PBS 1X, 3 μL of EthD-1 and 1.5 μL of calcein) was added on cells treated for 72 h with Biofiber (267 ± 53 µg) extract and on untreated cells (CTR).

Samples were incubated for 45 min in the dark condition, then the solution was removed, and cell nuclei were counterstained with 500 μL 4′,6-diamidino-2-phenylindole 1:100 (DAPI; Sigma Aldrich, Milan, Italy) for 10 min according to the protocol. Fluorescent image acquisition was carried out by semi-confocal microscope (ViCo confocal, Nikon). The experiment was carried out on three replicants.

### 2.10. Total RNA Extraction and Quantitative Real-Time PCR

For gene expression analysis, NHDF and HSF were seeded at a density of 5 × 10^4^ cells/well in 12-well cell culture plates (avantor VWR, Milan, Italy) and grown for 24 h in 2 mL DMEM to allow cell stabilization after seeding. 

Biofiber prototypes were cut in round samples with a diameter of 2 cm (762 ± 144 µg), sanitized under ultraviolet (UV) light for 24 h and fixed in respective well using sterile CellCrown™ inserts for 12 well plates to allow treatment. Untreated cells were used as the control (CTR). Cells were treated with Biofiber for 24–72 h in DMEM (2 mL); at specific experimental time, the inserts were removed, and cells prepared for RNA extraction.

Total RNA extraction was performed using 300 µL of Direct-zol RNA Miniprep’s reagents following the manufacturer’s protocol (Zymo Research; Euroclone S.p.A, Pero, Italy). Total RNA was then quantified by NanoDropTM (Thermo-Fisher Scientific, Milan, Italy) at 260 nm. A total of 300 ng of RNA was reverse transcribed using the iScript™ cDNA Synthesis Kit (Biorad Milan, Italy) and quantitative PCR analysis was performed using oligonucleotide primers displayed in Table 1. The reaction was carried out using MiniOpticon Real-Time PCR System (BioRad Laboratories, Milan, Italy) and data analysis was performed by CFX Manager Software. Gene expression was analyzed in triplicate and normalized to glyceraldehyde 3-phosphate dehydrogenase (GAPDH) gene expression using the 2-DDCT formula. 

The antifibrotic effect of Biofiber was evaluated observing the gene expression profile of relevant fibrotic markers (TGF-β1, α-SMA, TNF α; COL1A1, TGF-β1R1). The data are expressed as mean ± standard deviation (*n* = 3).

### 2.11. SDS–PAGE and Western Blot

Following the same culture protocol described in the gene expression paragraph, NHDF-treated and relative controls (CTR) were cultured for 24–72 h for COL1A1 and α-SMA protein level analysis by Western blot. The semi-quantification of these constitutional proteins was performed to confirm the antifibrotic prophylactic activity of Biofiber during the treatment period.

Briefly, at a specific experimental time, cells were scraped from the dish and lysed with ice-cold lysis buffer (100 mM NaHCO_3_, 1 mM EDTA, 2% SDS, 100 μM cocktail protease inhibitor, all from Sigma Aldrich, Milan, Italy) for 30 min on ice. The lysates were centrifuged at 13,000 rpm for 15 min at 4 °C, and supernatant protein concentrations were determined by Bicinchoninic acid assay (BCA, Pierce Thermo Fisher Scientific, Parma Italy) from a standard calibration curve prepared starting from a stock solution containing 2 mg/mL of bovine albumin. The stock solution was diluted with lysis buffer to obtain solutions of 15, 30, 60, 100, 150, and 200 μg/mL. Each standard solution was analyzed in triplicate, and each point of the calibration curve is the average of the three analyses (data not reported).

Equivalent samples (30 μg) were subjected to SDS-PAGE on 8% gel polyacrylamide; the proteins were then transferred onto Immun-Blot^®^ PVDF Membrane (BioRad Laboratories, Milan, Italy) and probed with primary antibodies anti-alpha-Smooth Muscle Actin (1:100; Cell Signaling Technology, Euroclone SpA, Pero, Italy), and Anti-Collagen I diluted 1:500 (Cell Signaling Technology, Euroclone SpA, Pero, Italy), followed by secondary antibodies conjugated to HRP (1:1000, Immunological Science, Rome, Italy).

Detection was performed with Enhanced Chemiluminescence (ECL) reagents (Cell Signaling Technology, Euroclone SpA, Pero, Italy) and revealed by autoradiography. The data are expressed as mean ± standard deviation (*n* = 3).

### 2.12. Statistical Analysis

The data are expressed as mean ± standard deviation (*n* = 3). Differences in mean values between the experimental groups were analyzed by two-way ANOVA (analysis of variance) followed by Tukey’s multiple comparison test using GraphPad Prism 7.0 software (Boston, MA, USA). Probability values * *p* < 0.05 were defined as significant, ** *p* < 0.01, *** *p* < 0.001, **** *p* < 0.0001 were defined as very significant.

## 3. Results

### 3.1. Polymer Solution Preparation and Characterization

The electrospinning technology is an emerging process for producing submicron polymeric fibers in the average diameter range from 100 nm to 5 μm [28]. Several studies have shown that fiber diameter has a strong relation with polymer solution viscosity and its concentration [29]. The design of Biofiber matrix fibers was set to obtain fibers with an optimal range of diameter to allow a persistent release of the antifibrotic agent and to promote exudate management for three days [27].

An amplitude oscillatory sweeps test was performed for assessing the viscoelastic properties and to determine the linear viscoelastic region of polymer solutions at different concentrations (0.1–20.0% *w*/*v*) loaded with the respective 2.0% *w*/*v* of NG. Results confirmed the viscoelastic liquid-like behavior of polymer solutions; LVER was found to interspace shear stress values from 1 Pa to 1000 (data not reported).

Through share ramp tests, it was possible to measure the intrinsic viscosity (*η_in_*) values of polymeric solutions (0.1–20.0% *w*/*v*) by the average share viscosity (*η*) reported in the LVER for each solution. 

**Figure 3:**
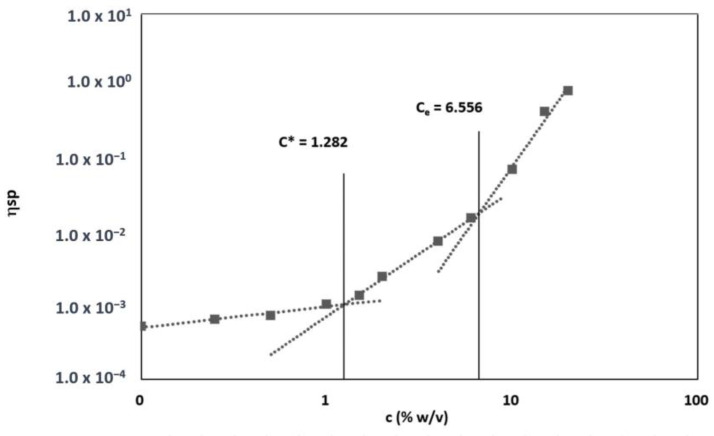
Specific viscosity dependence of specific (*η_sp_*) for Biofiber polymer solutions at 35 °C.

The viscosity concentration dependance was determined plotting specific viscosity value (*η_sp_*) vs. concentration (Figure 3). The different solutions of different concentrations can be separated into three regions: dilute, semi-diluted untangled, and semi-diluted entangled regime. At very low concentration (<1.0% *w*/*v*), PLA-PCL chains are dispersed separately, while as the concentration of polymer increases, the conformation of PLA-PCL chains start to overlap each other on the overlap concentration (c*), which is the crossover concentration between the dilute and semi-dilute regimes and equal to 1.282% *w*/*v*. With further increases in polymer concentration, an abrupt change in power law exponent for viscosity concentration dependence occurs, and the polymer chains begin topologically constraining each other and entangle in solution when entanglement concentration (C_e_) is reached. C_e_ is the crossover concentration between semi-diluted unentangled or semi-diluted entangled regions and represents the minimal polymer concentration required to obtain significant chain entanglement, and the value is 6.556% *w*/*v*; it followed that polymer concentrations >6.556% *w*/*v* are required to obtain suitable viscosity and superficial tension values that enable polymer chain entanglement and ensure a correct electrospun fiber generation (Figure 3).

### 3.2. Dressing Characterization 

#### 3.2.1. Morphology Characterization by Scanning Electron Microscopy

**Figure 4:**
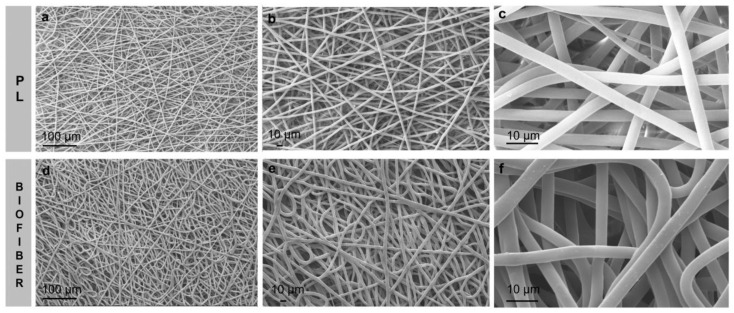
Representative SEM images of PL (**a**–**c**) and Biofiber (**d**–**f**) at different magnification 500×–1.0 K×–5 K×.

**Figure 5:**
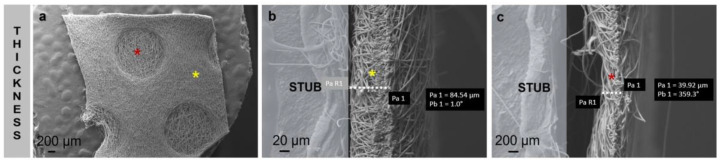
Representative SEM images of Biofiber texture electrospun dressing topography (**a**) at different magnification 50×–700×. Images of textured (red star), and no-textured area (yellow star) thickness measurement (**b**) and round pattern textured portions (**c**).

The data obtained by SEM analysis show smooth, bead-less fibers, and the matrix appears to be well interconnected with uniform and random fibers (Figure 4a–f). In general, no significant differences between placebo (PL) and Biofiber were observed and the addiction of NG in polymeric solution has not influenced the fiber morphologies (Figure 4d–f). The mean of fiber diameter was 3.825 ± 1.12 µm with a smooth surface. No evidence of NG crystals was highlighted by SEM analysis (Figure 4d–f), either in the electrospun matrices (Figure 5d,e) or on the nanofiber surface (Figure 4f). 

Electrospun matrix thickness was measured to highlight thickness value distribution and any structural defects because of textured round pattern (Figure 5a). The thickness of no-textured areas was 81.54 ± 0.21 µm (Figure 5b), greater than round textured regions that were 39.93 ± 1.02 µm (Figure 4c). No defect was highlighted in Figure 5a.

The round pattern was selected based on previous studies to improve the dressing performances in terms of mechanical properties and conformability to the body surface; moreover, this particular and bright texture contributes to the wound transpiration and modulates the exudate [16].

To further implement the breathability, the electrospinning process was set to contribute to the design of the matrix in terms of pore size and distribution, indeed the pores were distributed uniformly, and their size ranged between 70 and 90 μm^2^.

#### 3.2.2. Wettability Evaluation

Mepilex^®^Lite samples were found to share contact angle values of 93.43° ± 2.28° which is totally coherent with the field of application the medication is designed for. Biatain^®^ Alginate is associated with a null contact angle since the SWF drop was quickly absorbed by the alginate component of the dressing. Biofiber samples showed contact angle values of 109.3° ± 2.3 and PL patches of 108.88 ± 8.36, slightly higher than those of Mepilex Lite^®^ but in the same order of hydrophobicity, confirming the appropriate Biofiber wettability behavior; no statistically significant difference was highlighted between Biofiber and PL. A two-way ANOVA test was performed to evaluate data significance between samples and Mepilex Lite^®^.

#### 3.2.3. Fluid Handling Capacity

**Figure 6:**
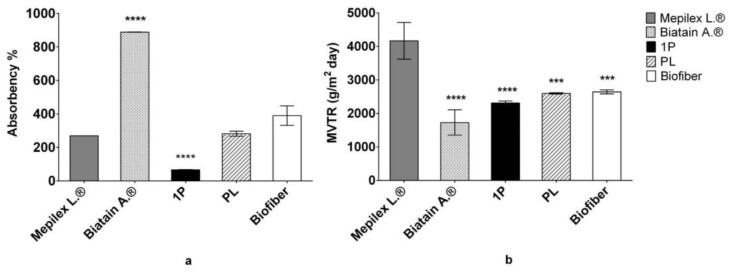
(**a**) Dressing absorption capacity (%), after 24 h of incubation in SWF (34 °C, RH Ambient). (**b**) Moisture vapor transmission rate (MVTR; 34 °C, 11% RH). Analyses were performed comparing placebo (PL) and Biofiber prototypes with no textured electrospun dressing (1P) and commercial controls (Mepilex Lite^®^ and Biatain^®^ Alginate). Statistically significant values are indicated as *** *p* < 0.001, **** *p* < 0.0001. Two-way ANOVA test was performed to evaluate data significance between samples and Mepilex Lite^®^. Some standard deviations are not noticeable as <10.

Fluid handling capacity (FHC), expressed as the sum of absorption rate and moisture vapor transmission rate (MVTR), indicates the dressing ability to manage the exudate (Figure 6a,b). This parameter has been evaluated by the BS EN 13726–1 standard test [16].

The production of wound exudate occurs because of vasodilation during the early inflammatory stage of healing under the influence of inflammatory mediators such as histamine and bradykinin [1]. A correct level of exudate is required to obtain physiological healing; in fact, if a wound bed becomes too dry, a scab will form that then impedes healing and wound contraction. The underlying collagen matrix and the surrounding tissue at the wound edge becomes desiccated. If a wound produces excessive amounts of exudate, the wound bed becomes saturated, and moisture leaks out onto the periwound skin causing maceration and excoriation [30].

Therefore, an innovative wound dressing must be able to absorb excess exudate and prevent the wound area from frying out [31].

##### Absorbency of Dressing

In the context of evaluating the exudate management properties of the electrospun matrices, contact angle measurements provide information regarding the expected tendency of the dressing to absorb exudate according to parameters including matrix porosity, polar chemical functions orientation, and fiber torsion, but do not inform about the actual performance of the matrix when exposed to experimental conditions mimicking the healing environment. The SWF uptake test was performed to evaluate the actual in vitro absorbency of the electrospun dressings; Mepilex Lite^®^, Biatain^®^ Alginate and no textured electrospun dressing (1P) were used as the controls.

The fluid handling capacity of all prototypes was evaluated at 24 h and results were compared to Mepilex Lite^®^.

Biatain^®^ alginate, due to its structures, has shown important absorption properties that have confirmed its important contribution to highly exude in wounds management [32].

Biofiber absorption capacity is totally comparable with Mepilex Lite^®^ results; this evidence confirmed that Biofiber corresponds to an optimal dressing for medium exuding wounds, and able to create a healing environment in wet conditions. Observing the no-textured electrospun dressing, used as one of controls, it is possible to appreciate how the circular texture improves the dressing absorbency performances, with a difference of ~140%. No significant differences were detected between the placebo (PL) and Biofiber (Figure 6).

##### Moisture Vapor Transmission Rate (MVTR)

Moisture vapor transmission rate (MVTR) is one of the basic physical properties of wound dressings that may influence the wound healing process in regulating the moisture microenvironment of wound healing; through MVTR, it is possible to evaluate the ability of a dressing to control water loss. An extremely high MVTR may increase the probability of eschar insurgence, whereas an unacceptably low MVTR may cause the accumulation of wound exudates and the maceration of the tissue. Hence, an ideal dressing with a suitable MVTR is required to provide a moist environment for establishing the best environment for natural healing. Previous studies determined that the dressing with an MVTR of ap-proximately 2028.3 g/m^2^ 24 h could maintain the optimal moisture content for the proliferation and function of epidermal cells and fibroblasts [33].

Data from the literature reported the MVTR of Mepilex Lite^®^ and Biatain^®^ Alginate to be 4170 ± 254.5 and 1730 ± 178.3 g/m^2^ day, respectively; regarding the no-texture electrospun dressing (1P), the MVTR was reported to be 2313.27 ± 58.86 g/m^2^ day.

**Figure 7:**
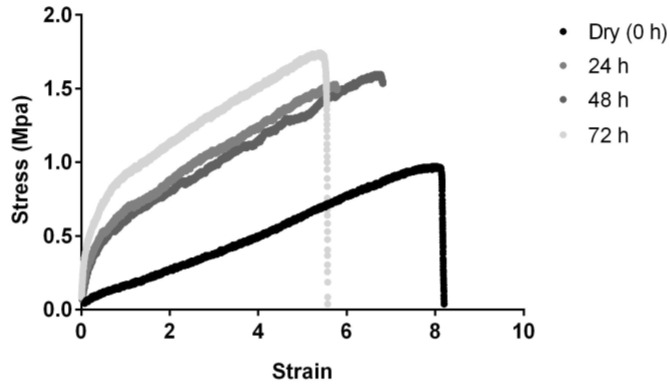
Representative tensile stress–strain curves of Biofiber prototypes in dry conditions (0 h) and after incubation in SWF (34 °C, 24–72 h, RH Ambient).

In this study, the MVTR value of Biofiber prototypes was 2645 ± 60.43 g/m^2^ day, demonstrating belonging to the optimal range of MVTR described in the literature. Its particular fiber composition gives it characteristics between the behavior of a dressing for poorly exuding wounds as Mepilex Lite^®^, and highly exuding wounds as Biatain^®^ Alginate (Figure 7b).

##### Vertical Wicking

Vertical wicking was selected as an additional test to furtherly evaluate the performance of the electrospun matrices in moisture management. The analysis was performed on the Biofiber prototypes, placebo (PL), and controls (1P, Mepilex Lite^®^, and Biatain^®^ Alginate).

Mepilex Lite^®^ and Biatain^®^ Alginate showed a vertical absorbent of 15 mm ± 0.33 and 31.6 mm ± 0.58 mm, respectively, confirming the moderate absorbent properties of the first and the maximum absorbent capacity of the latter.

Both NG-loaded and placebo electrospun samples were found to possess similar absorbent performance to Mepilex Lite^®^, resulting in 17.3 ± 2.93 mm and 15.72 ± 0.87 mm, respectively. No statistically significant result was detected between Mepilex Lite^®^ and both electrospun prototypes.

A two-way ANOVA test was performed to evaluate data significance between the samples and Mepilex Lite^®^.

#### 3.2.4. Mechanical Properties

The selection of the appropriate dressing is of paramount importance in wound management. The choice depends on the size, thickness, and location of the wound. Research is still ongoing to develop new and more advanced wound dressings, including smart polymeric bandages with ‘superior properties’ over existing commercially available dressings [33].

As reported in a previous study, the rounded pattern of Biofiber texture is optimal for flat surfaces which tend to suffer linear deformities such as in the shoulders, neck, and hips. Empty spaces provide perfect adaptability even for surfaces with concavity, such as the lower back [16].

In this study, Biofiber prototypes were evaluated at scheduled times (24–72 h) of incubation in SWF at 34 °C, RH Ambient to analyze mechanical properties’ variation between dry and wet conditions and over time of incubation.

A Biofiber stress–strain curve in response to constant tensile deformation rate highlighted the thermoplastic elastomeric behavior typical of amorphous materials as polymer based, with an optimal tenacity at all experimental times considered. Moreover, the stress–strain curve indicated an increment in tensile stress, and the respective decrease in matrix deformation, directly proportional to the time of incubation in the exudate (Figure 7).

Data in Table 2 reported the delta variation (ΔL%, variation between dry and wet prototypes) of Biofiber and Mepilex Lite^®^ mechanical properties in dry conditions and after 72 h of incubation in SWF.

Mepilex Lite^®^ presented optimal mechanical properties both in dry and wet conditions; unfortunately, being an adhesive polyurethane foam, this dressing does not achieve the ideal conformability to the body surface, and it tends to conserve its original shape under tensile stress (data not reported).

Analyzing the most significant Biofiber data it is visible that after 72 h of contact with exudate occurs an increase in the Young’s modulus and the respective decrease in elasticity by 38%; starting from an elongation of about 400% we are still in an excellent elongation range, even in wet conditions.

No data were reported regarding Biatain^®^ Alginate; the assessment of its mechanical properties was not completed due to the excessive tendency to reach the breaking point in response to tensile force.

#### 3.2.5. Dressing Integrity

The wound care practices represent one of the fundamental procedures that must be considered in the design and development of an innovative dressing. The dressing integrity is mandatory to allow a correct dressing removal procedure, free of events that can complicate the patient’s condition and additional pain.

The advanced medicated dressing was designed to persist on the wound site up to three days; for this reason, an accurate evaluation of its integrity was performed. No visual dispersion phenomena were observed for Biofiber and placebo (PL) prototypes. Results demonstrated that all formulations were stable over time (120 h), a minor mass loss below 8.0% was detected. 

**Figure 8:**
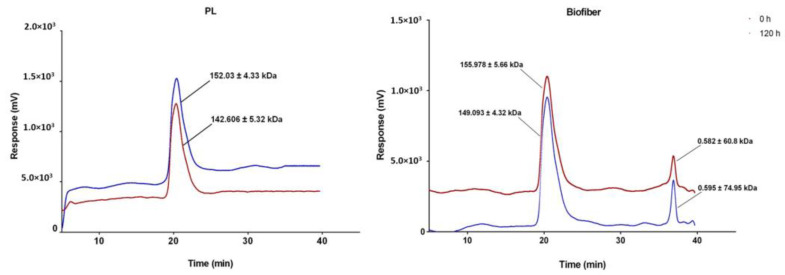
Chromatograms of placebo (PL) and Biofiber prototypes obtained through GPC analysis using isocratic grade THF at 0.8 mL/min as mobile phase. Red chromatogram (0 h) represents dry sample; blue signal (120 h) is referred to prototypes after 120 h of incubation in SWF at 34 °C, RH Ambient. Black lines indicate the respective molecular weight of PLA-PCL and NG before and after 120 h of incubation in SWF.

GPC data of dressing formulation after incubation in SWF at 34 °C, RH ambient for 120 h showed consistent data with a mass loss value with a slight reduction in molecular weight (2.85 ± 0.7%) for Biofiber prototypes and (5.16 ± 2.64%) for PL (Figure 8).

### 3.3. In Vitro Release Study

One of the biggest challenges in the field of innovative advanced dressings is to have a prolonged action on the application site, to avoid continuous dressing changes that can alter the granulation tissue in formation and cause pain to the patient [34].

The innovative polymer fibrous matrix was designed for the specific purpose of obtaining a 3-day controlled release of the encapsulated antifibrotic agent (NG 2.0% *w*/*w*), to improve wound care practices and patient compliance.

NG EE% calculated overall ranged between 100% and 96.47%. Total drug content, expressed as milligram per dressing (120 mg), was about 2.4 ± 0.7 mg/formulation.

The in vitro release test was performed to establish the amount of the antifibrotic agents released over the time. The study was conducted at scheduled experimental time points (2, 4, 6, 24, 48, 27 h) during incubation of Biofiber prototypes (2 cm diameter, 8.15 ± 0.54 mg) in PBS 1X, pH 7.4 at 34 °C in static conditions. 

**Figure 9:**
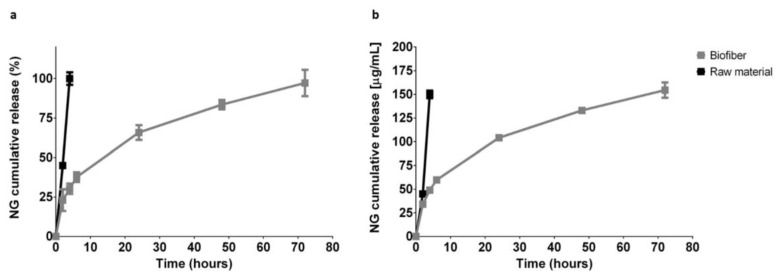
In vitro cumulative release expressed as (**a**) NG cumulative release (%) and (**b**) NG cumulative release [µg/mL] vs. time. Samples were incubated in PBS 1X, pH 7.4 at 34 °C, in static conditions. Dissolution of NG powder (Raw material) was used as control. In vitro release analyses were measured by UV–Vis spectrophotometer at 282 nm. Some standard deviations are not noticeable as <1.

The dissolution of NG powder (Raw material) was completed in four hours highlighting Biofiber capability to promote a sustained and controlled release for three days. The Biofiber formulations profile showed a modulated and gradual release of NG, almost complete at 72 h of analysis; the burst release at the sixth hour was at 11%. The low burst release could be attributed to the poor solubility of NG in aqueous solution as well as to the reduced amount of drug on the surface of the fiber. The cumulative release of NG (97.22 ± 8.35%) was detected at 72 h (Figure 9).

### 3.4. Cytotoxicity Assay

The MTT cytotoxicity assay was carried out at 24–72 h after treatment with extracts derived from Biofiber prototypes of different dimensions (0.25, 1, 2.25 cm^2^), to evaluate the viability (%) of NHDF/HSF (CTR+); NHDF/HSF treated with phenol (CTR−), and NHDF/HSF treated with Biofiber. All samples were analyzed in triplicate three times (N = 9). 

**Figure 10:**
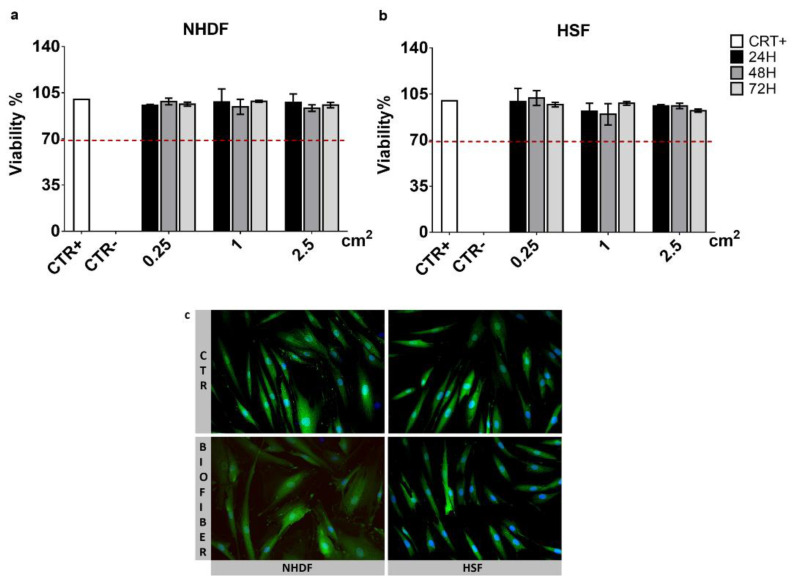
Indirect MTT cytotoxicity assay on NHDF (**a**), and HSF (**b**); Biofiber extracts (0.25, 1, 2.25 cm^2^) in DMEM (2 mL) at 37 °C for 24–72 h were used as the treatment solution. Untreated cells have been used as positive (CTR+) while, treated with phenol as negative control (CTR−). The viability threshold was fixed at 70% (red dot line) according to ISO 10993-12. The dressing safety was confirmed at 72 h of treatment, with live/dead staining (**c**). The observation will be performed with ViCo semi-confocal microscope (20×), whose function is managed by IMAJE PRO 6.2 software (Houston, TX USA). Some standard deviations are not noticeable as <10.

The results of cell viability were expressed as mean of cell viability % ± SD for samples. In both cell lines and in all experimental time points considered, the viability ranged from 85–100%, confirming the Biofiber biocompatibility; no dose–effect relationship was detected in all cases analyzed (Figure 10).

### 3.5. Live/Dead Staining

Live/dead staining was performed on NHDF and HSF at 72 h of treatment with Biofiber. In both cell lines considered, ~95% viability was detected. All values were >70% (viability threshold) according to ISO 10993-12, (Figure 10a,b). No evidence of cell damage or signs of apoptosis were detected (Figure 10c); the cell’s morphology was large, flat, and elongated (spindle-shaped) with processes extending out from the ends of the cell body. The cell nucleus was flat and oval. 

### 3.6. Gene Expression Analysis by Quantitative Real-Time PCR

Gene expression analysis was evaluated through qPCR at 24–72 h of treatment to validate the antifibrotic effect of Biofiber on NHDF and HSF; all results were compared with the respective untreated cells. Data are representative of three independent experiments.

These two different cell lines were selected to assess the ability of prototypes to constrain HTS formation in an early wound healing process and in a process already assessed. 

**Figure 11:**
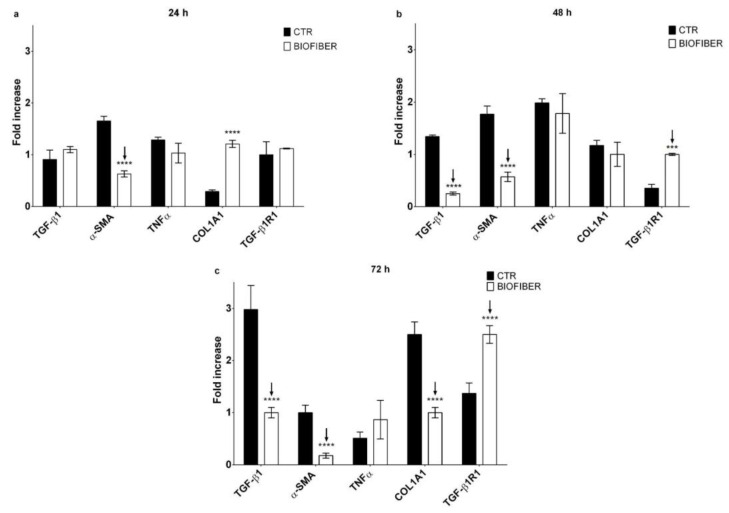
Gene expression analysis of NHDF treated with Biofiber for 24, 48, and 72 h. (**a**) qRT-PCR at 24 h. (**b**) qRT-PCR at 48 h. (**c**) qRT-PCR at 72 h. Results are normalized to the housekeeping gene (Glyceraldehyde-3-Phosphate Dehydrogenase (GAPDH)). Statistically significant values are indicated as *** *p* < 0.001, **** *p* < 0.0001. Analysis of variance test was performed to evaluate data significance.

**Figure 12:**
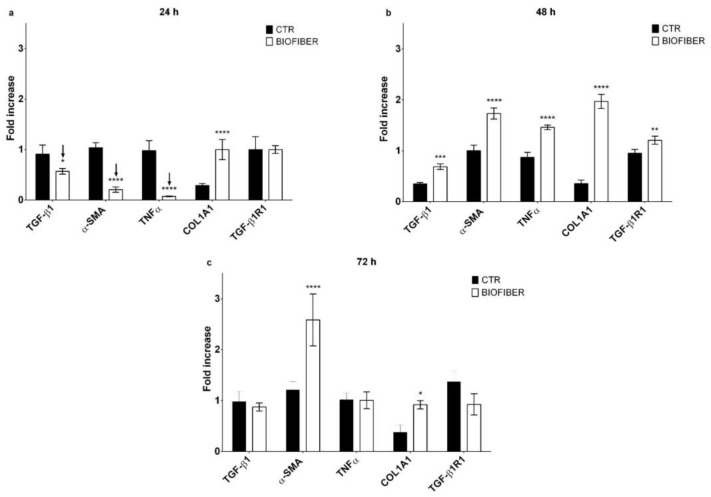
Gene expression analysis of HSF treated with Biofiber for 24, 48 and 72 h. (**a**) qRT-PCR at 24 h. (**b**) qRT-PCR at 48 h. (**c**) qRT-PCR at 72 h. Results are normalized to the housekeeping gene (Glyceraldehyde-3-Phosphate Dehydrogenase (GAPDH)). Statistically significant values are indicated as * *p* < 0.05, ** *p* < 0.01, *** *p* < 0.001, **** *p* < 0.0001. Analysis of variance test was performed to evaluate data significance.

The expression levels of fibrotic genes such as TGF-β1, α-SMA, TNF α, COL1A1, and TGF-β1R1 were detected by RT-qPCR normalized by the GAPDH gene expression (Figure 11 and Figure 12).

Regarding the NHDF treated for 24 h with Biofiber, α-SMA was expressed as 1.02-fold lower than CTR (Figure 11a, *p* < 0.0001); no other significant antifibrotic gene downregulation was detected. 

After 48 h, the expression of TGF-β1, and α-SMA was respectively 1.09 and 1.19-fold lower in NHDF treated with Biofiber (Figure 11b, *p* < 0.0001). Regarding TGF-β1R1 the expression in treated cells was 0.65-fold higher than CTR (Figure 11b; *p* < 0.001); thus, after two days of treatment, the effect of Biofiber seems to enhance a preliminary antifibrotic action on NHDF with the significant downregulation of the major fibrotic gene and the physiological increment of TGF-β1R1 one.

In the last phase of treatment, the gene expression of TGF-β1 and α-SMA in NHDF was further modulated by Biofiber antifibrotic action. The expression of TGF-β1, and α-SMA were, respectively, 2.88 and 0.85-fold lower than untreated cells. In addition to the evidence obtained at 48, after 72 h of treatment the expression of COL1A1 was 1.55-folder lower than CTR. The TGF-β1R1 level was 1.13-fold higher, thus confirming the physiological increment of this gene because of low levels of the relative ligand (Figure 11c; *p* < 0.0001). 

**Figure 13:**
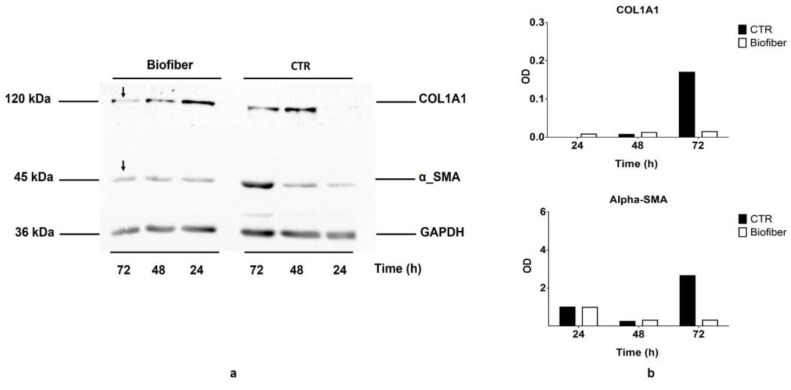
(**a**) Representative Western blot results of COL1A1 (120 kDA) and α-SMA (42 kDa) in NHDF untreated and treated for 24–72 h with Biofiber; black arrows indicate relevant results. (**b**) Graphical representation of COL1A1 and α-SMA blot bands of plot a normalized against total GAPDH expression.

The gene expression analysis of HSF treated for 24 h with Biofiber (Figure 12a) showed a significant antifibrotic effect with the downregulation of TGF-β1 and α-SMA; the modulation of gene expressions was, respectively, 0.34 and 0.83-fold lower than CTR levels. The treatment with Biofiber for 24 h has also showed the modulation of inflammation in HSF, the expression of TNFα was, indeed, 0.92-fold lower than CTR (Figure 13a, * *p* < 0.05, *** *p* < 0.001, **** *p* < 0.0001).

No significant antifibrotic and anti-inflammatory effect was detected in HSF treated with Biofiber advanced dressing for 48 and 72 h (Figure 12b,c).

### 3.7. SDS–PAGE and Western Blot

About one week after dermal injury, the provisional matrix is replaced by granulation tissue, a neo-formed connective tissue composed of small vessels, high levels of extracellular matrix, and fibroblastic cells that become activated and modulate into myofibroblasts; all these elements are under the influence of the increment of TGF-β1 level. The main feature of myofibroblasts is represented by an important contractile apparatus like that of smooth muscle and by the neo-expression of α-smooth muscle actin (α-SMA), and hypersecretion of COL1A1 [35].

The antifibrotic action of Biofiber prototypes loaded with (NG 2% *w*/*w*), was also assessed evaluating the downregulation of the protein levels of COL1A1 (120 kDa) and α-SMA (42 kDa) through a Western blot assay. The analyses were performed on NHDF treated with Biofiber for different scheduled times (24–72 h), and on NHDS-untreated cells as the control (CTR). The results reported in Figure 13a show a time-dependent modulation of both protein levels. COL1A1 and α-SMA blot bands were normalized against total GAPDH expression. The most relevant result was detected after 72 h of treatment with the modest decrease in COL1A1 and α-SMA protein levels. In addition, an indicative densitometry analysis was performed to give a graphical representation of downregulation trend over time (Figure 13b). On the bases of these preliminary results, it is possible to assume that the treatment with Biofiber could promote the modulation of α-SMA and COL1A1 protein levels, during all phases of the early wound healing process.

## 4. Discussion

NG-loaded PLA-PCL electrospun fibers have been produced by electrospinning technology. Previous studies revealed that PLA and PCL complement each other in terms of in vitro performances when used as a blend or copolymer [36,37]. Moreover, the PCL homopolymers increase the adhesiveness of fiber to human tissue, indicating that PCL polymer and related copolymers (as PLA-PCL) are more suitable in wound dressing applications [38,39]. The success of PCL-based products in wound healing is apparent from the many recent patents and encouraging reviewers, in general. Valuable knowledge and patent data provide an important contribution to strategic research and development, product prototyping and acquisition, and patent licensing [36].

A comparative analysis was carried out among Biofiber (placebo electrospun dressing), Biofiber NG (electrospun dressing containing NG 2% *w*/*w*), Mepilex Lite^®^ and Biatain^®^ Alginate (as commercial polyurethane-based and alginate-based dressings, respectively), and NG solution [37]. Exudate management, mechanical properties, and prophylactic treatment results were used as criteria assessment. Biofiber and Biofiber NG protypes have shown values of wettability, absorbency, MVTR, and vertical wicking such as with Mepilex Lite^®^. The latter is suggested for moderate exudate wounds. Regarding NG solution, the exudate management feature was not detected due to the limit of the dosage form. Biatain^®^ Alginate advanced dressing has showed a more hydrophilic behavior with a non-detectable wettability and high percentage of SWF absorbency according to its hydrophilic polymeric composition. As already mentioned in the introduction, an ideal advanced dressing must provide a correct moisture balance to create a healing in a humid condition and prevent the risk of maceration and eschar formation [9,10]. Based on this evidence, Biofiber and Biofiber NG provides optimal exudate management performance.

Mechanical properties are one of the major challenges in the design of conformable and flexible dressing; tensile strength and elongation are fundamental to achieve the complete adaptability to the body surface both in dry and wet conditions. Biofiber and Biofiber NG have shown optimal mechanical properties during a 3-day test, especially for the elongation and yield strength. Mepilex Lite^®^ clearly ensured the highest mechanical properties even though it is characterized by poor conformability when it is applied to a concave surface (data not reported). No mechanical results were delivered for Biatain^®^ Alginate and NG solution because of their poor mechanical properties and dosage form, respectively. Optimal integrity and suitable biocompatibility have been demonstrated for both Biofiber and Biofiber NG.

The in vitro cumulative release studies validated the ability of the PLA-PCL electrospun fibers to provide a sustained controlled release of NG for three days; these features along with the exudate management and the dressing stability confirmed its sustained functional performances on the wound bed. PCL polymer and related copolymers have been widely exploited for the delivery of therapeutic agents and tissue engineering and have been tested in several new drug delivery systems [38,39]. In this analysis, the unicity of Biofiber NG in the prophylactic treatment of HTSs is clear. Biofiber regulates the gene expression of the major scarring genes through an NG-controlled release for 72 h. The extended release of Biofiber allows to reduce dressing changes and consequently cutting down healthcare costs and implementing patient compliance. No sustained antifibrotic effect was shown by the NG solution according to the conventional dosage form (solution) requiring multiple administrations. No antifibrotic effect was detected with the other treatments (Mepilex Lite^®^ and Biatain^®^ Alginate) in accordance with the company’s statement.

Promising results regarding the reduction of α-SMA and COL1A1 constitutional protein levels were obtained only at 72 h; this time lag between gene expression and protein level results was due to the delay among gene transcription and protein translation. The analysis of protein levels was possible only in a representative manner as it was limited by the in vitro model experimental timing; considering the structural role of both these proteins, we can assume that a probable solid decrement will be achieved during a continued Biofiber application throughout the wound healing process. Y. Song et al. stated that NG may be a new drug for the treatment of hypertrophic scars, their data demonstrated that NG solution at different concentrations (10–40 µmol) inhibits the scarring, at least to some extent, through its inhibition of Akt^p−Ser473/Thr308^ [40]. These data are in accordance with more recent research on induced mouse models of skin fibrosis [41].

The antifibrotic activity was not detected on HSF where no relevant result was observed. Further in vivo investigations are required to confirm these preliminary results, and to exceed the limits given by in vitro-cell cultures that have not allowed to assess the antifibrotic efficacy over a prolonged period. As reported in HTS gene expression analysis, the efficacy of this advanced medicated dressing was not achieved in a scar process already concluded. Considering the fibrotic process, this is modulated by high levels of constitutional elements; for this reason, it is difficult to knock down the expression at both gene and protein levels of these structures once consolidated. In 2015, it was demonstrated that NG ointment exerts its wound healing effect by providing a positive effect on wound side re-epithelization; however, no evidence of NG antifibrotic activity was identified [42]. In addition, to the best of our knowledge, no evidence is reported in the literature regarding the lack of efficacy of naringin treatment when a skin scar process is already established.

## 5. Conclusions and Future Prospective

Exploiting the variety of physical, chemical, and mechanical properties offered by electrospinning technologies, the present work demonstrated the scientific contribution that Biofiber could apply in the complex wound care field. The advantages of this innovative prophylactic treatment are enclosed in its innovative textured fibrous matrix that offers conformability and resistance to the application site, and the ability to reach the prolonged effect of the antifibrotic agent at the site of action for three days, to reduce damage to the granulation tissue and pain, improving healing and patient compliance. Its point of excellence lies in the prevention activity; Biofiber, applied in the early stages of wound healing, performs a prophylactic effectiveness that makes it advantageous compared to conventional and invasive treatments.

Currently, ex vivo studies on healthy human skin models are in progress to evaluate the efficacy on the connective reorganization of the healing tissue and understand the action in a complex environment; these studies, combined with upcoming in vivo accurate studies on appropriate scarring experimental models, are needed to fully validate the effectiveness of Biofiber as a topical prophylactic antifibrotic agent. 

## Figures and Tables

**Table 1:** Summary of primers used for quantitative PCR analysis.

Title 1	Primer Forward	Primer Reverse
H. Sapiens TGF-β1	5′-GGACCAGTGGGGAACACTAC-3′	5′-GGCATGGACTGTGGTCATGA-3′
H. Sapiens α-SMA	5′-GCAGCCGAGCCAAGCACTGT-3′	5′-TGGGAGCATCGTCCCCAGCA-3′
H. Sapiens TNF α	5′-CAATCGGCCCGACTATCTCG	5′-GCCGTTTGGGAAGGTTGGATG-3′
H. Sapiens COL1A1	5′-CTGCCTGGTGAGAGAGGTC-3′	5′-CACGATGACCACGACGGC-3′
H. Sapiens TGF-β1R1	5′ATTGCTGGACCAGTGTGCTT -3′	5′-ATGGTGAATGACAGTGCGGT-3′
H. Sapiens GAPDH	5′-TTCACCACCATGGAGAAGGC-3′	5′-GGCATGGACTGTGGTCATGA-3′

**Table 2:** Mepilex Lite^®^, Biatain^®^ Alginate, and Biofiber ΔL% of dry vs. wet conditions (SWF, 34 °C for 72 h). Statistically significant values are indicated as * *p* < 0.05, ** *p* < 0.01, *** *p* < 0.001, **** *p* < 0.0001. A two-way ANOVA test was performed to evaluate data significance.

Samples	ΔL%				
	Elongation	UTS	Breaking Point	Yield Strength	Young’s Modulus
Mepilex Lite^®^	26.17 ± 8.84	305.14 ± 54.10 ****	272.33 ± 7.49 ***	0.58 ± 0.07	202.9286 ± 50.85 *
Biatain^®^ Alginate	-	-	-	-	-
Biofiber	28.18 ± 1.51 **	26.64 ± 14.66	-3.077 ± 1.58	0.41 ± 0.089	2661.70 ± 890.37 ****

## Data Availability

Data will be made available on request.
